# Circulating Tumor Cells: From Theory to Nanotechnology-Based Detection

**DOI:** 10.3389/fphar.2017.00035

**Published:** 2017-02-01

**Authors:** Yue Ming, Yuanyuan Li, Haiyan Xing, Minghe Luo, Ziwei Li, Jianhong Chen, Jingxin Mo, Sanjun Shi

**Affiliations:** ^1^Department of Pharmacy, Institute of Surgery Research, Daping Hospital, Third Military Medical UniversityChongqing, China; ^2^Key Laboratory for Stem Cells and Tissue Engineering, Ministry of Education, Sun Yat-sen UniversityGuangzhou, China; ^3^Department of Histology and Embryology, Zhongshan School of Medicine, Sun Yat-sen UniversityGuangzhou, China

**Keywords:** cancer stem cells, stem-cell properties, circulating tumor cells, metastasis, nanotechnology, CTC isolation and detection

## Abstract

Cancer stem cells with stem-cell properties are regarded as tumor initiating cells. Sharing stem-cell properties, circulating tumor cells (CTCs) are responsible for the development of metastasis, which significant affects CTC analysis in clinical practice. Due to their extremely low occurrence in blood, however, it is challenging to enumerate and analyze CTCs. Nanotechnology is able to address the problems of insufficient capture efficiency and low purity of CTCs owing to the unique structural and functional properties of nanomaterials, showing strong promise for CTC isolation and detection. In this review, we discuss the role of stem-like CTCs in metastases, provide insight into recent progress in CTC isolation and detection approaches using various nanoplatforms, and highlight the role of nanotechnology in the advancement of CTC research.

## Introduction

Tumors are heterogeneous tissues composed of abundant phenotypically and functionally distinct cell subpopulations each having different capacities to grow, differentiate, develop drug resistance and form metastases. Stem cell properties, including self-renewal, the capability to develop into multiple lineages, and the potential to proliferate extensively, play critical roles in the generation of complex multicellular organisms. A growing body of evidence suggests that stem cell properties are relevant to some forms of human cancer, and cells with these properties are involved in tumor metastasis and drug resistance (Fang et al., 2005). Cancer cells sharing stem cell properties are called “cancer stem cells” (CSCs). CSCs are intrinsically drug resistant and often evade chemotherapies, and they eventually induce tumor relapses or metastases (Malanchi et al., 2012). During metastatic dissemination, circulating tumor cells (CTCs) invade distant organs and settle in supportive niches (Pantel et al., 2009). In this process, the stem cell-like properties within CTCs contribute to CTC survival and eventually seed the growth of a secondary tumor. It is documented that high CTC numbers indicate poor prognosis even after effective radiotherapies and chemotherapies. Monitoring CTC levels may aid in predicting the response to ongoing therapy and developing personalized medicine.

Based on these considerations, the CTC enumeration and detection shows great clinical value, and their prognostic significance has been demonstrated in several types of cancers, including breast, prostate, colon, melanoma, and lung cancer (Masuda et al., 2016). However, due to extreme rarity, it is challenging to detect and analyze CTCs. Therefore, an ideal technology with great efficiency and sensitivity, which can also release and collect captured CTCs with a high vitality, should be developed for CTC studies. To date, a vast number of isolation and detection techniques have been developed based on the inherent properties of CTCs (big size, for example), but none of the methods are perfect because of issues of insufficient capture and low purity. The development of a better method of detection and treatment of the rare and important CTCs is desirable.

During the last decades, nanotechnologies have been rapidly developed, and examples of nanotechnology-based approaches to improve CTC detection have accumulated. A variety of advanced nanomaterials have been applied to CTC enrichment and detection, such as nanoparticles. With the large surface-to-volume ratio, nanomaterials enable highly efficient cellular binding in the complex blood matrix. In addition, CTCs naturally prefer nanostructural surfaces due to their similar scales on the surface of cells. Furthermore, nanomaterials functionalized with antibodies can enhance CTC capture efficiency and specificity. In this review, we discuss the role of stem-like CTCs in metastases. We then provide insight into recent progress on CTC enrichment and detection approaches using various nanoplatforms and highlight the role of nanotechnology in the advancement of CTC research.

## Circulating Tumor Cell Theory

**FIGURE 1:**
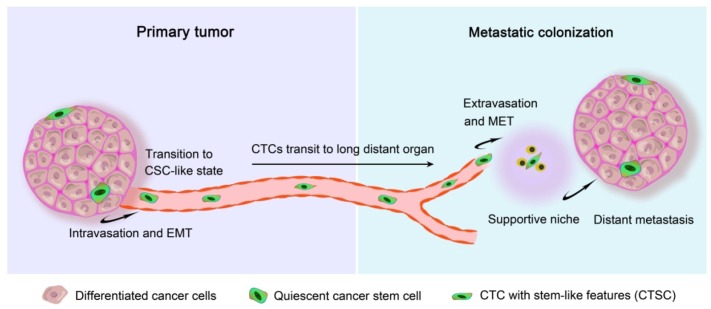
**Metastatic colonization based on circulating tumor stem cells**. Metastasis proceeds through multiple steps that occur in two major phases: (i) the pre-colonization phase of metastasis comprises physical translocation of cancer cells from the primary tumor to the circulation and (ii) the colonization phase after circulating tumor cell (CTC) extravasation. Cancer cells within the primary tumor undergo an epithelial-mesenchymal transition (EMT) process and endow invasive capacity, then intravasate into the tumor vasculature in the form of CTCs, which must be able to survive in the circulating blood and evade from the innate immune response and other defenses. Once CTCs home to a secondary site, the settlement in supportive niches enables them to survive and retain their stem-like tumor-initiating capacity. These cancer cells then enter a latent state lasting from months to decades while they adapt to their newfound microenvironment. When this latency is broken out, the cancer cells reinitiate overt outgrowth and overtake the local tissue microenvironment to commence the coming colonization.

The blood of many patients with advanced primary carcinomas contains CTCs, which can transit to distant organs to form future metastases after adaptation and proliferation. These CTCs are the cells sloughing from the edges of a primary tumor mass and intravasating to enter hematogenous circulation or the lymphatic system, and can remain loose in circulation, cluster together as they travel, or lodge themselves in new tissues (Williams, 2010). Since in 1869, CTCs were first detected in cancer patients, and the studies on the role of CTCs in cancers are attractive. It was demonstrated that the presence of five or more CTCs (breast, prostate, and lung cancer), and three or more CTCs (colorectal cancer) per 7.5 mL of peripheral blood caused a shorter median progression-free survival and shorter overall survival, suggesting that the number of CTCs before treatment is an independent predictor of progression-free survival and overall survival (Cristofanilli, 2004). Therefore, it can be concluded that high CTC numbers are closely correlated with increased tumor aggressiveness (Pavese and Bergan, 2014) and metastasis (Cohen et al., 2008; de Bono et al., 2008), which lead to poor prognosis even after effective radiotherapies and chemotherapies. Recently, scientists have generated patient-derived xenograft experimental models of breast (Baccelli et al., 2013), lung (Hodgkinson et al., 2014), and prostate cancer (Williams et al., 2015) by using CTCs, demonstrating the tumor-initiating capacity of CTC in metastasis. CTCs create a successful metastatic deposit involving a cascade of linked sequential steps: once lodged in a foreign tissue, CTCs may invade the local host tissue, survive to proliferate, and eventually seed a secondary colony (**Figure 1**). Thus, the proliferative and self-renewal abilities, which are regarded as the main stem cell-like properties, are critical for CTCs to facilitate tumor metastasis in foreign tissues. It was validated that stem cell markers are frequently overexpressed in CTCs, and therefore, metastasis may be evoked by subpopulations of CTCs that express CSC markers (Aktas et al., 2009). These CTCs with stem cell-like properties are considered as a “seed” for secondary tumors.

As a source of metastatic cells, CTCs could become not only a potent prognostic marker to indicate therapy effectiveness or necessity even in the absence of detectable metastases but also an integral part of tumor staging criteria which are currently focused on the primary tumor (Cristofanilli et al., 2005; Pierga et al., 2012). Moreover, CTCs coming from cancer patients provide an opportunity to study patterns of drug susceptibility (Yu et al., 2014), indicating that it may be a novel potential target for tumor treatment. In light of these considerations, as well as the simple and minimally invasive process of blood collection, CTC analysis could be used as a real-time “liquid biopsy” for patients with cancer. However, there are significant technical challenges that impede CTC isolation and detection owing to the rarity of CTCs in blood. Therefore, a better understanding of CTCs may make their clinical use more applicable.

### The Stem Cell-Like Properties within CTCs

Cancer stem cells are malignant cells with the capacity of self-renewal, the potential to develop into any cell in the overall tumor population, and the proliferative ability to drive continued expansion of the population of malignant cells (Jordan et al., 2006). It is widely accepted that CSCs play a critical role in cancer initiation, progression, recurrence, and metastasis (Nguyen et al., 2012; Shi et al., 2013). CSCs, possessing elevated tumor-initiating ability (Pattabiraman and Weinberg, 2014), can be identified by biomarkers (regarded as stem-like markers) including epithelial cell adhesion molecule (EpCAM), CD44, CD24, CD133, and aldehyde dehydrogenase (ALDH), etc. (Ajani et al., 2015; Lu et al., 2016). Moreover, CSCs display the ability to self-renew, which is often implicated in several signaling pathways, such as Wnt, Notch, and Hedgehog (Hh) signaling pathways (Takebe et al., 2011).

Evidence for stem-like CTCs existence was convincingly documented based on the expression of stem cell markers in CTCs (**Table 1**). ALDH1 is a classical stem cell marker that is frequently expressed in CTCs from patients with breast cancer (Reuben et al., 2011; Kasimir-Bauer et al., 2012), pancreatic cancer (Ting et al., 2014), and lung cancer (Hanssen et al., 2016). Theodoropoulos et al. (2010) showed that 35.2% of CTCs in metastatic breast cancer patients had the CD44^+^/CD24^-/low^ phenotype, which is highly associated with stemness and tumorigenic potential. CD133, an identified CSCs indicator, was overexpressed in CTCs and contributed to resistance to cancer therapies in breast cancer (Nadal et al., 2013) and colorectal cancer (Iinuma et al., 2011). Schölch et al. (2016) reported that the stem cell marker Bmi1 was significantly upregulated in CTCs, suggesting that CTCs possess a stem cell-like phenotype and an increased capacity of self-renewal and tumor formation. Moreover, EpCAM, which is considered a CSC marker, is often expressed in CTCs and commonly used for their enrichment and detection. Furthermore, the tumor-initiating capacity of CTCs was proven through xenografts from freshly isolated CTCs. Hodgkinson et al. (2014) enriched EpCAM^+^cytokeratin^+^ CTCs from the blood of small-cell lung cancer (SCLC) patients and subcutaneously injected them into immunocompromised mice to form palpable tumors within 4 months. Blood samples from metastatic breast cancer patients were depleted of hematopoietic cells and transplanted into the femoral medullar cavity of immunocompromised mice to test whether CTCs are tumorigenic. Within 6–12 months after transplantation, EpCAM^+^CD44^+^CD47^+^MET^+^ CTCs developed multiple bone, lung, and liver metastases in mice (Baccelli et al., 2013). In addition, a xenograft model was successfully generated with CTCs from isolated prostate cancer patients (Williams et al., 2015).

**Table 1:** Stem cell markers in CTCs of cancer patients.

Marker	Type of cancer	No. of patients	Marker expressed (%)	Analytical methodology	Reference
EpCAM	Breast cancer	24	83.3%^∗^	Flow cytometry	Hyun et al., 2016
	Head and neck cancer	15	40%^∗^	CellSearch system	Nichols et al., 2012
ALDH	Breast cancer	92	46%^∗^	AdnaTest	Kasimir-Bauer et al., 2012
	Colorectal cancer	27	33%^∗^	PCR	Gazzaniga et al., 2010
	Breast cancer	24	54%^#^	RT-PCR	Barrière et al., 2012
CD133	Breast cancer	23	30.1%^#^	Triple fluorescence	Bock et al., 2015
	Prostate cancer	20	100%^∗^	Flow cytometry	Nadal et al., 2013
CD44	Colorectal cancer	150	40%^∗^	RT-PCR	Katoh et al., 2015
	Breast cancer	24	33%^#^	RT-PCR	Barrière et al., 2012
CD24^-/low^	Breast cancer	20	35.2%^#^	Immunofluorescence microscopy	Theodoropoulos et al., 2010
Bmi1	Lung cancer	10	70%^∗^	RT-PCR	Ana et al., 2016
	Breast cancer	24	67%^#^	RT-PCR	Barrière et al., 2012

^∗^*Among CTC positive patients; ^#^among CTCs captured from patients*.

### CTCs and Metastasis

Due to shed off from primary tumors followed by intravasation into the circulation system, cancer cells with stem-like properties performed as circulating tumor stem cells (CTSC) (Clevers, 2011) (**Figure 1**). In the early steps of the metastatic cascade, CTCs have been thought to be involved the epithelial-mesenchymal transition (EMT) process (Aceto et al., 2014), which leads epithelial cells to weaken their cell–cell adhesion and gain migratory and invasive properties, thus becoming mesenchymal-like cells (Ksiażkiewicz et al., 2012). Once induced in cancer cells, EMT may allow them to escape from the primary tumor, invade into the circulation, and reach the site of future metastasis (Krawczyk et al., 2014). Upon arriving at their destinations, the interactions between such CTCs and the local microenvironment or “niche” modulate tumor metastatic colonization (Li et al., 2007). The survival and stemness of CTCs can similarly benefit from interactions with supportive niches, which are rich in developmental and self-renewal signals, such as Hh, Wnt, and Notch (Massagué and Obenauf, 2016). Factors secreted by stromal cell can also influence the stem cell niche, providing a suitable microenvironment for cancer initiation and development. For example, breast carcinoma clones are primed for metastasis in the CXCL12-rich microenvironment of the bone marrow (Zhang et al., 2013), while bone morphogenic proteins (BMPs) can induce CD133^+^ CSCs to differentiate and markedly attenuate the tumor-forming ability of CD133^+^ cells (Visvader and Lindeman, 2008). Due to protection by the niche, cancer cells are able to adapt to the microenvironment, initiate proliferation and eventually seed a successful metastasis. Finally, CTCs usually undergo a process of mesenchymal epithelial transition (MET) and recover their epithelial character to proliferate and differentiate into different secondary or metastatic tumors (Nieto, 2013). EMT is now known to not only facilitate the metastatic spread and the progression of cancer cells (Mitra et al., 2015), but also to support the induction of a stem cell phenotype with properties such as self-renewal associated with high invasiveness and resistance to apoptosis and therapy (Mani et al., 2008). Stem-like CTCs may proliferate as tumor-initiating cells to form aggressive tumors during relapse/metastasis.

### CTC Characterization

Circulating tumor cells often occur at very low concentrations in the blood, with estimates of just one CTC per ∼10^7^ white blood cells (WBCs) per milliliter of blood, suggesting that any assay for CTC enumeration must be able to handle many surrounding/normal cells and the error rates will be high. Therefore, the different properties of CTCs that distinguish them from the surrounding normal hematopoietic cells can be utilized for their enrichment and detection. Generally, CTCs have large size distributions of 20∼30 μm in diameter, which is much larger than that of capillary bores (∼8 μm) (Chaffer and Weinberg, 2011). However, their physically plastic properties facilitate the pass of CTCs into circulation, and indeed CTC clusters can rapidly and reversibly reorganize into single-file chain-like geometries and transit through capillary-sized vessels (Au et al., 2016). In addition, CTCs can be cloaked by platelets or by coagulation factors to protect themselves from the immune system and oxidative stress (Labelle et al., 2011; Le et al., 2015). Thus, it is extremely difficult to accurately detect CTCs. However, CTC-based biomarkers provide useful approaches for capturing and detecting CTCs.

EpCAM frequently overexpressed on the surface of CTCs can be used as a cell biomarker (Maetzel et al., 2009; Huch and Dollé, 2016). It has demonstrated that CTCs show a high level of expression of EpCAM in numerous human malignancies (Went et al., 2006; Barsan et al., 2013). Yamashita et al. proved that EpCAM^+^ CTCs are an independent risk factor for HCC recurrence (Yamashita et al., 2013; Zhou et al., 2016). Consequently, anti-EpCAM is usually used for CTC screening. However, EpCAM is expressed in not all tumors, and absent EpCAM expression has been cited as a reason for the lack of CTC capture (Khan et al., 2011; Mikolajczyk et al., 2011). In addition, cytokeratins (CKs), including CK8, CK18 and CK19, are largely associated with CTCs, and fluorescent anti-CKs antibodies are often used to recognize and mark CK-positive cells.

### Current Advances in the Isolation of Circulating Tumor Cells

Certainly, CTCs can provide an ideal approach to strongly affect cancer diagnosis and treatment strategies. Therefore, the detection of CTCs in blood samples is becoming one of the most active areas of translational cancer research. To improve the detection efficiency and speed, various sophisticated systems have been developed, which generally rely on the different properties and characteristics between WBCs and CTCs within the blood (Ee et al., 2016). Currently, methods based on physical properties, antibodies and nucleic acid are used to isolate and detect CTCs from whole blood samples. For instance, the large size, mechanical plasticity and dielectric properties of CTCs may endow their significant distinction compared with normal cells (Gascoyne and Shim, 2014; Sollier et al., 2014; Park et al., 2016). These physical properties are logically utilized for the isolation and enrichment of CTCs which are fast, simple and label-free; however, these methods are restricted by their non-specificity, which can be overcome by an antibody-combined method (Arya et al., 2013). Antibody-based functional assays include immunocytochemistry, immunomagnetic, and adhesion-based methods. Presently, the CellSearch assay, involving an immunomagnetic technique and image cytometry, is the only FDA-approved CTC diagnostic technology for metastatic breast, prostate, and colorectal cancer (de Wit et al., 2014). The sensitivity and specificity of markers are critical for the efficiency of such techniques, although none of the markers are expressed exclusively. Thus, two or more main approaches can be generally envisioned for this purpose. In nucleic acid-based approaches, CTCs can be detected using DNA- or RNA-based technologies with impressive sensitivity. For example, AdnaTest kits (AdnaGen, Germany), which utilize the technique of multiplex reverse transcription PCR (RT-PCR), allow simultaneous amplification and detection of multiple transcripts of circulating DNA or RNA (Markou et al., 2011). Additionally, several other methods, such as microscopy imaging, CTC filters, acoustic-based separation, etc., are available to detect CTCs (Zheng et al., 2011; Lopez-Riquelme et al., 2013; Li et al., 2015). Even though there are various approaches that have been developed for the detection of CTCs, none are ideal to meet the application needs, as issues of CTC loss, low purity, and narrow detection spectrum still need to be addressed (Viswanath and Kim, 2016). Most of these methods contain a series of complicated processes, such as erythrocyte lysis, cell centrifugation and washing, which may lead to insufficient capture and even a waste of detection time and money. Overall, because of the extreme rarity of CTCs and inadequate sensitivity and specificity, CTC detection and enumeration is still not a part of routine tumor staging in clinical practice.

The challenge of CTC detection is related to the requirement of high sensitivity combined with high specificity (Paterlini-Brechot and Benali, 2007), however, several factors still hinder the analysis of CTCs which can be summarized as: (i) CTCs are very rare in the circulation, (ii) there is no marker that can reliably and efficiently distinguish these CTCs from other blood-borne cells (Esmaeilsabzali et al., 2013) and (iii) downstream molecular and genomic characterization are challenging due to the low number of cells that can be isolated (Friedlander et al., 2014). To date, the CTC analysis techniques should be urgently developed with high sensitivity and selectivity, which can also be used for the rapid purification of CTCs, paving the way for downstream CTC characterization, and obtaining viable CTCs for *ex vivo* expansion (Lin et al., 2014). Currently, nanotechnology provides extensive applications in biology and medicine, and many researchers take advantage of nanotechnology to improve the efficiency and sensibility of CTC capture and to accelerate detection speed. Nanomaterials possess many unique and excellent physical properties that can be used to overcome the limitations of traditional CTC detection methods and make viable CTCs more accessible.

## Nanotechnology in CTCs

Nanotechnology has made excellent contributions to tackle oncology over the past several decades. The uniquely appealing features of nanotechnology for drug delivery, diagnosis and imaging facilitate its application in cancer (Shi J. et al., 2016). For example, nanoparticles possess greater surface areas and more functional groups that can be linked with multiple diagnostic and therapeutic agents (He L. et al., 2016). In cancer therapy, nanotechnology has enabled the development of targeted drug delivery, enhanced the properties of therapeutic molecules, and sustained or stimulus-triggered drug release (Shi S. et al., 2016). In addition, the development of tumor-targeted contrast agents based on nanotechnology may offer enhanced sensitivity and specificity for *in vivo* tumor imaging, which is able to detect solid tumors, determine recurrence, and monitor therapeutic responses (Wang et al., 2008).

Despite being perceived as one of the most promising developments in the treatment of cancer, nanotechnology in the detection and therapy of CTCs leaves plenty of room for improvements, especially for the targeting ability. Nanotechnology offers a fundamental advantage for early detection, accurate diagnosis, and personalized treatment of malignant tumors. In CTC isolation and detection, it can predominantly improve their efficiency and sensitivity. Also, nanotechnology can carry drugs and provide approaches for CTC target treatment. In this review, we would provide insight into recent advances in CTC detection and therapy achieved through nanotechnology applications.

Nanomaterials may provide access to improve the enrichment of extremely scarce CTCs, making the counting and analyzing of CTCs more precise (Xiong et al., 2016). For instance, with the advantage of facilitating of cellular internalization, magnetic nanoparticles (MNPs) can be utilized to enrich and detect cancer cells under magnetic microarray condition. Nanoroughened surfaces, as well as nanopillars, nanowires, and nanofibers, possess large specific surface areas that can increase interactions with extracellular features. In addition, carbon nanotubes (CNTs) and graphene oxide (GO) can enable electrical conductivity to access sensing functionality (Yoon et al., 2014). More importantly, a certain number of CTCs are known to be lost due to the lack of specificity in these methods. Therefore, nanomaterials functionalized with various antibodies were carried out to target CTCs. EpCAM antigen is frequently used as a target for CTC enrichment, as it was widely expressed on the cell surface of CTCs derived from carcinomas and not detected on blood cells (Allard and Terstappen, 2015). With the rapid development of technology, the combination of nanotechnology with these specific antigens will provide promising approaches for CTC isolation and enumeration.

### Immunomagnetic Nanobeads

Immunomagnetic technology is used extensively in CTC enrichment and detection, because it is easy to manipulate and exhibits high capture efficiency and specificity. Based on antibody-antigen binding, immunomagnetic technologies have good sensitivity that makes it especially suitable for rare CTC separation. Additionally, in immunomagnetic assays, a magnetic field can be introduced without direct contact with cells and attract cells over a broader spatial domain (Chen et al., 2013). Thus far, various types of immunomagnetic technologies for CTC separation have been invented. In the earlier stage, magnetic particles (microbead) were in range more than 0.5 μm, while MNPs emerged with a smaller diameter in 5–200 nm (Bhana et al., 2015). MNPs commonly composed of magnetic elements, such as cobalt (Co) and iron (Fe), show alignment of their magnetic moment in the presence of magnetic field. MNPs reveal higher cellular binding capability and excellent stability in whole blood. Their smaller size makes the attachment to CTCs with many MNPs easy and leads to a higher magnetic susceptibility. Furthermore, MNPs with various biomarkers can be exploited to characterize CTCs.

**FIGURE 2:**
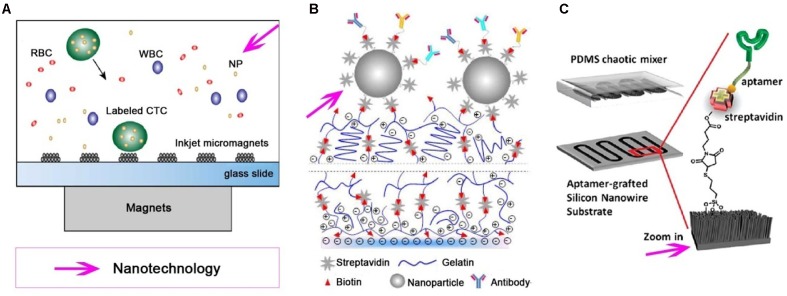
**Various nanotechnologies utilized for isolation and detection of CTCs**. Different nanotechnologies, such as magnetic nanoparticles (MNPs), nanostructured substrates and/or combine with a microfluidic chip, are developed for effective and specific isolation and detection of CTCs. **(A)** Immuno-magnetic microchip for the detection of CTCs labled with MNPs, which pre-functionalized with anti-epithelial cell adhesion molecule (anti-EpCAM) (Chen et al., 2015). **(B)** A nanostructured coating contained nanoparticle can detect and release CTCs from peripheral blood (Eduardo et al., 2015). **(C)** An aptamer-grafted silicon nanowire substrate (SiNS) covered by a polydimethylsiloxane (PDMS) chaotic mixer is exploited as a microfluidic CTC chip (Zhao et al., 2016).

CellSearch system, a commercially available device for CTC detection, uses Fe_3_O_4_ MNPs coated with anti-EpCAM antibody to confer magnetic properties to the EpCAM positive cells, resulting in magnetic separation of CTCs from the bulk of other cells in the blood (Truini et al., 2014). The CellSearch system can enumerate CTCs as low as two CTCs in 7.5 mL of peripheral blood (Allard et al., 2004). MNPs functionalized with anti-EpCAM antibodies were used to bind selected cells in the presence of a magnetic field in a reversibly bonded nanotextured polydimethylsiloxane (PDMS) chamber using NdFeB block magnets (Hoshino et al., 2011). Kim et al. (2013) presented a microseparator for the isolation of CTCs using immunomagnetic nanobeads with bound EpCAM antibodies, revealing that the CTC microseparator isolated approximately 90% of spiked CTCs in human peripheral blood and purified to approximately 97%. In another micromagnet array, which was fabricated integrating inkjet-printing technology combined with immunomagnetic microchip, the whole blood sample is labeled with MNPs and separated due to the magnetic momentum when the mixture flows through the microchannel (**Figure 2A**) (Chen et al., 2015). Immunomagnetic nanospheres (IMNs) fabricated by a convenient and highly controllable layer-by-layer assembly method successfully captured more than 94% of tumor cells in whole blood via only a 5 min incubation (Wen et al., 2014). These efforts of using immunomagnetic nanotechnology as a CTC capture platform promoted their potential use in clinic. Meanwhile, criticism is often expressed regarding the fact that detecting only EpCAM^+^ cells via this platform would miss some EpCAM^-^ CTCs and yield a false negative detection (Andree et al., 2015).

### Nanostructured Substrates

Nanoscale components present in the tissue microenvironment, including the extracellular matrix (ECM) and cell-surface structures, have been documents. Due to their mutual scale as a fundamental asset, nanostructured substrates with nanoscale topography that mimic the natural ECM or basement membrane can effectively interact with cell surface components. The major feature of nanostructured substrates is their larger biomimetic surface area. This feature not only enhances the interaction between substrates and cell surface targeting but also benefits the isolation and detection of CTCs (Wang et al., 2013). Furthermore, nanostructured substrates can be coated with higher densities of ligand and then increase the binding affinity to cells (Bhana et al., 2015). Therefore, nanostructured substrates with improved enrichment and detection accuracy may be developed as promising bio-platforms for CTC detection.

Various nanostructure substrates have been employed for CTC detection, including nanopillars (Wang et al., 2011), nanowires (Shen et al., 2013), nanofilms (Wang et al., 2016), nanocoatings (**Figure 2B**) (Eduardo et al., 2015), and PDMS (Islam et al., 2015), etc. Silicon nanopillars (SiNP) with diameters in the range of 100–200 nm on a silicon wafer have been used for cancer cell isolation, and the capture yield of MCF-7 cells in culture medium was 45–65% on SiNP; 10 times more than what was achieved on flat silicon (Wang et al., 2009). Tseng and colleagues pioneered a concept of “NanoVelcro” cell-affinity substrates, in which CTC capture agent-coated nanostructured substrates were utilized to immobilize CTCs with a high efficiency. Generations of NanoVelcro CTC chips based on silicon nanowire substrate (SiNS) were developed with a high CTC capture efficiency (Wang et al., 2011; Liu and Wang, 2014; He W. et al., 2016). The thermoresponsive NanoVelcro chip was created by grafting thermo-responsive polymer brushes [poly (*N*-isopropylacrylamide), PIPAAm] onto SiNS and incorporating with an overlaid PDMS chaotic mixer. This chip can increase the contact frequency between CTCs and NanoVelcro substrates, as well as the capture and release of CTCs with fast temperature responsiveness at 37 and 4°C, respectively (Ke et al., 2015). Furthermore, the CTCs can be purified and subjected to amplifications of their genomic DNA. Recently, a degradable zinc-phosphate-based hierarchical nanosubstrate (HZnPNS) (400–800 nm) emerged for the capture and release of CTCs (Shan et al., 2016). HZnPNS was functionalized with anti-EpCAM antibody and detected 6–75 CTCs/mL from metastatic cancer patient blood samples, which outperformed CellSearch system (1–125 CTCs/7.5 mL). In addition, HZnPNS allowed 88 ± 4% of captured cells to be gently released with a high viability of 92 ± 1%. Although nanostructured substrates can be used as an ultrasensitive tool for enriching rare CTCs from blood samples and keeping most captured cells viable for subsequent molecular analysis, further testing on clinical samples is necessary before reaching conclusions. There is still a challenge for purification platforms to pave the way for subsequent molecular and functional analysis.

### Incorporation of Nanostructures in Microfluidics

Microfluidics, another recent technological development, can also improve the capabilities of CTC capture and detection. In microfluidics systems, low volumes of fluids are processed to achieve multiplexing capabilities and high-throughput screening (Volpatti and Yetisen, 2014). Taking advantage of small sample-volume requirements, fast processing times and large surface areas, various microfluidic platforms have been developed for rare CTC capture and enrichment (Hajba and Guttman, 2014). Various cell separation mechanisms have been devised in microfluidic platforms, including mechanisms that rely on magnetic forces, affinity chromatography, size and/or deformability-based isolation, and dielectrophoresis (Peng et al., 2013). Microfluidic technology is of great promise for detecting viable CTCs with satisfactory efficiency and purifying CTCs for down-stream biology analyses (Trifanny et al., 2016).

Recent progresses in nanotechnology have aided the development of advanced CTC-detecting microfluidic devices for the recovery and purification of CTCs. Immunomagnetic assays are often combined with microfluidic technology to enrich and detect CTCs. MNPs are commonly used to label target tumor cells. When blood sample flows through the microchannel on top of an array of permanent magnets, nanoparticle-labeled cells can be separated and captured on the substrate of the microchannel. The average capture rates for SkBr3, PC3, and Colo205 cells were 97, 107, and 94%, respectively (Huang et al., 2013). A nanoelectronic microfluidic chip fabricated by silicon nanograss (SiNG) electrodes was developed for the label-free distinguishing of both epithelial and mesenchymal CTCs (Hosseini et al., 2016). This system showed a great preference for MCF-7 and MDA-MB231 cancer cells with high capture yields between 92 and 97%. In another recent report, SiNS was embedded in the microfluidic chip (**Figure 2C**) in the combined use of rationally designed aptamer cocktails to achieve enhanced and differential capture of CTCs from non-small cell lung cancer (NSCLC) patients through a synergistic effect (Zhao et al., 2016). Kwak et al. (2017) utilized MNPs functionalized with anti-EpCAM antibodies to integrate the surface of the Mag-Gradient Chip based on microfluidics. The simple and multi-functional Mag-Gradient Chip can isolate 3 mL of heterogeneous CTCs sample in 1 h and realize 95.7% EpCAM-positive and 79.3% EpCAM-negative CTC isolation (Kwak et al., 2017). Although advances in microfluidic technologies are likely to accelerate CTC detection, many challenges hinder the clinical applications of CTC technologies.

### Nanotechnology in Stem-Like CTC Detection

CD44, which generally acts as a cell surface hyaluronic acid (HA)-binding glycoprotein, is frequently exploited to target CSCs. Prospectively, CD44 has also been investigated to identify stem-like CTCs. Galanzha et al. (2009) developed a photoacoustic (PA) and photothermal (PT) flow cytometry platform for *in vivo* detection and killing of CTCs with a stem-like phenotype (CD44^+^ CTCs). MNPs functionalized with anti-CD44 were used to bind and capture the stem-like CTCs under a magnet, and golden CNTs (GNTs) conjugated with anti-CD44 were exploited to kill the stem-like CTCs at a low confluence. Magnetic-induced clustering of MNPs within single cell, yielded 6.6-fold enhanced PT signals compared to the intact area before magnetic action. Then, the PT technique was applied to demonstrate ablation of the CD44^+^ CTCs labeled by GNT-CD44, confirmed by cell membrane damage and changed in the optical and fluorescent images. Recently, Hong et al. (2016) reported a multifunctional magnetic nanowire (NW) offering a significant improvement in CTC isolation efficiency. On these magnetic NWs, five different types of antibodies, including DAPI, anti-EpCAM, anti-CD44, anti-vimentin, and anti-CD45, were employed to differentiate CTCs from the surrounding leukocytes. In particular, anti-CD44 was employed to capture CTCs with stem-like properties. Furthermore, anti-CD44, combining anti-EpCAM, anti-Keratin18, and anti-insulin-like growth factor antigen (anti-IGF-I), was also used to functionalize silver-coated gold nanorods (AuNR/Ag) as surface-enhanced Raman scattering (SERS) multispectral contrast agents for CTC detection (Nima et al., 2014).

Although nanomethods are certainly capable of identifying stem-like CTCs through surface stem cell markers, there is a lack of nanomethods capable of capturing individual stem-like CTCs with a high sensitivity. Because none of these stem cell markers can be used exclusively to isolate CTCs in every type of tumor, a combination of multitype antibodies might help to specifically target CTCs. In addition, newer isolation and detection nanotechnologies such as nanostructured substrates and/or the incorporation of microfluidics devices might achieve that level of sensitivity. Another critical need is the development of nanotherapeutics for the stem-like CTCs. Beyond their highly sensitive nano–bio interactions, drug carriers with nanotechnologies may be potentially developed for the delivery of various anticancer therapeutic agents to kill stem-like CTCs.

### Other Nanotechnologies in CTC Detection

A new SERS nanoparticle system was proposed for the direct detection of CTCs in the blood (Wu et al., 2015). The hydrodynamic diameter was determined to be 21 nm for SERS nanoparticles. Under the optimized experimental conditions, these SERS nanoparticles exhibited excellent specificity and high sensitivity for the direct detection of cancer cells in rabbit blood. The limit of detection (LOD) was 5 cells/mL, and a good linear relationship was obtained between the SERS intensity and the concentration of cancer cells in the range of 5–500 cells/mL, which demonstrates that the SERS nanoparticles can be used for the quantitative analysis of cancer cells in the blood. Overall, although further testing of clinical blood samples is necessary, SERS nanoparticles are promising for use in the direct detection of CTCs in blood with excellent specificity and high sensitivity.

Carbon nanotubes have remarkable electronic properties and have been applied for the electric detection of cancer cells in the blood. Yang et al. (2014) demonstrated a quantitative CNT-based sensor for direct detection of cancer cells in whole blood using real-time electrical impedance sensing. Multi-walled CNTs (MWCNTs) are used to increase the surface area and electrical conductivity of sensors. The binding of tumor cells to EpCAM antibodies causes increased electron-transfer resistance. The detection limit of the proposed system for the target cancer cells is at a minimum concentration of 10 cells/mL in whole blood samples. This method, via the simple process of fabricating the sensors, accurate structural control over the electrode surfaces, and high sensitivity and specificity for the target cells, provides a promising platform for the early diagnosis of cancer. In addition, Banerjee et al. (2015) reported a non-invasive strategy for isolating cancer cells by autonomously propelled CNT microrockets. This designed microrocket had the ability to rapidly target (∼5 min) and efficiently capture (∼85%) TfR^+^ cancer cells coming from an artificial CTC-like suspension, then it can also function as a magnetic isolation platform to capture cells from peripheral blood cells following high resolution imaging. As a result, it may provide an approach for the rapid and efficient extraction of CTCs.

## Conclusion and Prospects

Cancer stem cells are believed to be responsible for tumor relapse and metastasis, and stem cell properties are involved in CTCs, known as stem-like CTCs, which contribute to seed a distant metastasis. As CTC levels in the blood of patients can allow for the early detection of cancer and act as a prognostic indicator, the analyses of CTCs to screen patients for the presence of metastatic disease is of great value in clinical practice. Despite its clinical relevance, the isolation and detection of CTCs can be quite challenging due to the extremely rare presence of CTCs within the blood. To date, nanomaterials have emerged with many excellent physical properties, including the nanoscale size which is similar to ECM, large specific surface area increasing the interaction with the target cells. What’s more, nanomaterials can be functionalized with antibodies to specifically recognize cells. Taking advantage of these properties, increasing nanotechnologies have been developed as a coordinator to improve the sensitivity and efficiency of CTC isolation and detection and achieving the release of CTCs, such as immunomagnetic nanobeads and nanostructure substrates. Nanotechnologies are also incorporated in microfluidics devices because it is more difficult to use one method for all types of cancer. Even if the commercial success of CTC detection can be achieved in the clinic, the survival or quality of life does not seem to be improved. The major reason for this can be concluded as the lack of anti-CTC therapy activities in these methods. That is, patients should be undergo targeted CTC therapy to eliminate CTCs in the peripheral blood after CTC detection.

## Author Contributions

YM summarized the literature and wrote the manuscript. YL helped with the manuscript writing. HX revised the manuscript. ML wrote part of the manuscript. ZL prepared table and figures. JC and JM revised the manuscript and provided critical comments. SS supervised all the works and wrote the manuscript.

## Conflict of Interest Statement

The authors declare that the research was conducted in the absence of any commercial or financial relationships that could be construed as a potential conflict of interest.
